# Deciphering the mechanism of light quality regulating the quality of sun-cured yellow tobacco based on GC-MS non-targeted metabolomics

**DOI:** 10.3389/fpls.2026.1770112

**Published:** 2026-04-22

**Authors:** Lijuan Chen, Jinxiu Zhang, Guirong Su, Xiangli Xie, Xiaoqing Shi, Lu Zhang, Weiai Zeng, Jie Yu, Xixin Zhou, Yi Zhang

**Affiliations:** 1 College of Agronomy, Hunan Agricultural University, Changsha, Hunan, China; 2 College of Biological Science and Technology, Hunan Agricultural University, Changsha, Hunan, China; 3 Tobacco Production Technology Center, Hunan Tobacco Company, Changsha, Hunan, China

**Keywords:** differential aroma metabolites, light quality, metabolomics, quality, sun-cured yellow tobacco

## Abstract

Sun-cured tobacco is cured by means of natural sunlight, a process that is highly susceptible to fluctuations in external environmental conditions, hindering standardized production and consequently leading to poor quality consistency. Therefore, light is a key environmental factor regulating the curing process of sun-cured tobacco, and as a core component of light, light quality can directly regulate the synthesis and accumulation of metabolites within sun-cured tobacco leaves, thereby governing the formation of their aromatic properties and core quality traits. However, existing studies have rarely explored the mechanisms underlying how light quality regulates aroma formation and core quality in sun-cured tobacco, leaving the intrinsic regulatory mechanisms largely unclear. To address this knowledge gap, the present study investigated how light quality regulates the aroma of sun-cured tobacco. Using the ‘Cun Sanpi’ variety, we applied three light treatments during the curing stage: natural light (NL), NL supplemented with red light (NL+R), and NL supplemented with blue light (NL+B). We analyzed leaf quality and metabolic dynamics through conventional chemical analysis, sensory evaluation, and GC-MS non-targeted metabolomics. The results indicated that light quality significantly altered tobacco quality. The NL+R treatment led to increased sugars, decreased nicotine, low mucosa irritation, and a pleasant aftertaste. In contrast, the NL+B treatment resulted in higher nitrogen and nicotine levels, which corresponded with poorer sensory quality. The NL treatment yielded leaves of intermediate quality. Metabolomic analysis identified 40 differential aroma-related metabolites, primarily carbohydrates and organic acids. These were significantly enriched in 23 metabolic pathways, with core pathways involving carbohydrate metabolism, amino acid metabolism, and secondary metabolite biosynthesis. We found that light quality drives differential metabolite accumulation by modulating metabolic rhythms and specific pathways: NL+R activated ubiquinone and terpenoid-quinone biosynthesis; NL+B regulated glycolysis/gluconeogenesis and propanoate metabolism; and NL influenced photosynthesis. This study identifies key metabolites, specific pathways, and their correlation with sensory traits in light-quality-mediated regulation of sun-cured tobacco. Our findings provide a theoretical foundation for optimizing light conditions during curing to improve tobacco quality in a targeted manner.

## 1 Introduction

Tobacco is not only a vital global industrial crop but also a model plant for elucidating fundamental processes such as photosynthesis and secondary metabolism, holding irreplaceable value in both agricultural production and life science research (Li et al., 2025). Among tobacco varieties, sun-cured tobacco has emerged as a raw material of great application value in cigarette formulations due to its complex aroma profile, pronounced fragrance, and low tar delivery (Sun et al., 2018). Consequently, the formation mechanism of its flavor quality has garnered extensive attention. Notably, the development of these core quality traits in sun-cured tobacco is highly dependent on environmental conditions during the curing process, with light being the paramount factor. Light exposure is essential in the curing process of sun-cured tobacco and represents the most distinct difference compared to the curing methods of flue-cured tobacco. It not only shapes the quality traits of sun-cured tobacco (Guangdong et al., 2015; Jingjing et al., 2018) but also serves as a pivotal signal, directly regulating the accumulation of metabolites in these plants (Yang et al., 2022).

Existing studies have confirmed that light of different spectral qualities (e.g., red, blue, ultraviolet, and infrared) can regulate plant physiological metabolism across multiple dimensions—including photosynthetic efficiency, carbon-nitrogen balance, and secondary metabolite synthesis—through specific wavelength combinations (Wu et al., 2025). This characteristic makes light a potentially effective tool for the targeted improvement of tobacco quality. However, significant differences exist in the responses of different tobacco types to light quality, and these differences are closely associated with their distinct curing methods, genetic backgrounds, and metabolic characteristics. For flue-cured tobacco, which is cured under dark and temperature-controlled conditions, the regulatory effects of light quality are mainly concentrated in the pre-curing. By influencing the synthesis of key aroma precursors such as chlorophyll, carotenoids, and neophytadiene, light quality further modulates the smoothness and aroma richness of the final products (Zhen et al., 2023). In contrast, sun-cured tobacco is exposed to direct light throughout the entire curing period, which renders the regulatory role of light quality in its metabolic processes more comprehensive and dynamic. For sun-cured tobacco, the dynamic accumulation and transformation of various metabolites(e.g., carbohydrates, organic acids, amino acids, alkaloids, and phenols) (Liu et al., 2022), during the curing process form the core material basis for its aroma profile development. Furthermore, aroma quality is a key determinant of the market value of tobacco products. Research has shown that light quality can regulate the accumulation of aroma-related metabolites in flue-cured tobacco and Oriental tobacco. For instance, red light promotes the synthesis of sugars and terpenes (Sankhuan et al., 2022; Mokhtari et al., 2025), while blue light enhances the biosynthesis of organic acids.

The results of sensory evaluation are a direct reflection of the commercial value of sun-cured tobacco, with its key metrics being closely related to the composition and content of volatile metabolites in the leaves. Specifically, degradation products of carbohydrates, such as pyrazines, serve as key precursors for sweet and baked aromas (Mortzfeld et al., 2020; Zhang et al., 2025). Volatile compounds like phenolics and terpenoids directly contribute to the fresh and floral characteristics of the tobacco (Liu et al., 2023), while changes in the content of alkaloids and their derivatives significantly influence the mucosa irritation and smoothness of the smoke (Li Z. et al., 2024). Therefore, the conventional chemical components of sun-cured tobacco indirectly shape its sensory quality by regulating the synthesis and transformation of volatile metabolites. Consequently, differences in the volatile metabolite profile form the core link connecting physiological metabolic changes under light quality regulation to the final sensory attributes. Clarifying this hierarchical relationship among the three is a crucial prerequisite for deciphering the mechanism by which light quality regulates the formation of aroma quality in sun-cured tobacco.

The extraction and detection of aroma compounds in tobacco leaves typically involve techniques such as solid-phase extraction, liquid-phase microextraction, and headspace extraction (Ding et al., 2013), coupled with analytical instruments like liquid chromatography—tandem mass spectrometry (LC-MS/MS) and gas chromatography—mass spectrometry (GC-MS) (Cai et al., 2002).Traditionally, targeted analytical methods based on GC-MS or LC-MS are primarily used for the precise quantification of one or a few known chemical components (e.g., polyphenols and alkaloids) in tobacco. However, these approaches lack the comprehensiveness needed to fully characterize complex aroma profiles (Zheng et al., 2019). In contrast, untargeted metabolomics—while also employing platforms such as GC-MS and LC-MS—adopts a non-presumptive, global analytical strategy. It enables the simultaneous detection and comparison of thousands of metabolites, thereby systematically revealing the overall chemical basis of tobacco aroma. This approach compensates for the limitations inherent in traditional targeted methods (Patti et al., 2012).

Our previous research has observed that sun-dried yellow tobacco treated with different light qualities (such as natural light, supplementary red light, and supplementary blue light) shows significant differences in conventional chemical composition and sensory evaluation quality. This strongly suggests that light quality may influence the formation of the final aroma quality of tobacco leaves by regulating their metabolic processes. However, current research still has obvious deficiencies: most works only focus on a single category of metabolites or short-term time-quality responses, and fail to comprehensively analyze the dynamic metabolic mechanism of aroma formation mediated by light quality from the perspective of the entire curing cycle. More importantly, the core metabolites that cause the differences in the above-mentioned chemical components and sensory qualities, the metabolic pathways specifically regulated by light quality, and the intrinsic correlation between metabolic changes and sensory characteristics remain unclear. This greatly limits the precise application of light quality regulation technology in the production of sun-dried yellow tobacco.

Based on this, the present study aims to systematically elucidate the metabolic mechanisms through which light quality regulates aroma formation in sun-cured tobacco. Using differences in conventional chemical composition and sensory quality of sun-cured tobacco under different light treatments (natural light, natural light+ red light, and natural light+ blue light) as a starting point, we employed GC-MS-based untargeted metabolomics to investigate the restructuring of the metabolic network during the curing process. By integrating multi-dimensional data, this study is designed to address three core objectives (1): to identify the key metabolites responsible for quality differences under different light regimes (2); to reveal the specific metabolic pathways regulated by distinct light qualities; and (3) to elucidate how these metabolic changes ultimately link to sensory characteristics. By focusing on the dynamic metabolic processes throughout the curing cycle, this study employs untargeted metabolomics to systematically decipher light quality-mediated metabolic regulatory mechanisms and construct a complete “light quality-metabolic pathway-sensory quality” regulatory chain for sun-cured tobacco. It is expected to address the limitations of existing research and provide a more comprehensive theoretical basis for the targeted regulation of aroma quality in sun-cured yellow tobacco. The findings are expected to provide a solid theoretical foundation for optimizing light-environment management strategies and for the targeted enhancement of aroma quality in sun-cured tobacco production. Furthermore, this work holds significant practical value for promoting sustainable tobacco production and the development of high-value products.

## 2 Materials and methods

### 2.1 Materials and processing

Mature leaves of the “Cun Sanpi” variety, cultivated in Wanfu Village, Zifu Town, Ningxiang City, Hunan Province (a typical growing area for sun-cured tobacco in Ningxiang), were selected as experimental materials. A standardized sampling protocol was followed during harvesting. Tobacco plants with consistent growth and uniform agronomic traits in the field were selected, and their upper middle leaves (specifically, leaves positioned at nodes 15–22 counting from the base) were used. The specific harvest criteria were as follows: the main vein appeared milky white, the leaf surface was yellowish-green, mature yellow spots covered 30–40% of the leaf area, and the leaf margins were slightly curled. All samples were collected on sunny days between 9:00 and 11:00 a.m. to avoid interference from dew. After harvest, the leaves underwent vein scoring and folding operations before being transferred to an experimental greenhouse (30 m in length, 8 m in width, and 3.1 m in height) for curing.

Three treatment groups and one initial control group (CK 0d) were designed in this experiment. The treatment groups included NL (natural light only, no supplementary light), NL+R (natural light + supplementary red light, 670 nm), and NL+B (natural light + supplementary blue light, 460 nm). The control group (CK 0d) consisted of fresh, un-cured tobacco leaves sampled directly from tobacco plants. Supplementary lighting was implemented on the 1st, 2nd, and 3rd days of the curing process (yellowing stage), from 7:00 a.m. to 7:00 p.m. daily for the NL+R and NL+B groups; the NL group was maintained under natural light conditions without artificial supplementation. No supplementary lighting was provided for any group after the 3rd day of curing. A schematic timeline depicting the full curing cycle (including the yellowing stage, color-fixing stage, and stem-drying stage) is presented in (**Supplementary Figure 1**) to clarify the chronological sequence of the experiment. Sampling was conducted at three critical time points, as specified below:Time point 1: Fresh tobacco leaves without any light supplementation or curing treatment were collected and used as the initial control samples for the experiment.Time point 2: On the 1st, 2nd, and 3rd days of curing (yellowing stage), representative leaves were separately collected from the three treatment groups (NL, NL+R, and NL+B), with 6 biological replicates set for each group. Time point 3: On the final day of curing (Day 9), representative leaves were collected again from the aforementioned three treatment groups, with 6 biological replicates also set for each group. Immediately after sampling, the tobacco leaves were flash-frozen in liquid nitrogen and transferred to a -80 °C ultra-low temperature freezer for storage until GC-MS metabolomics analysis. The entire sampling process was conducted under strict aseptic operation norms to prevent sample contamination.

### 2.2 Measurement Indicators and methods

#### 2.2.1 Metabolomics detection

In this study, metabolomic profiling was conducted using a gas chromatography–mass spectrometry (GC-MS) platform. The sample pretreatment procedure for metabolite extraction and derivatization was as follows: approximately 50 mg of each sample was accurately weighed into a 2 mL centrifuge tube. Then, 0.5 mL of a methanol–water solution (CH₃OH:H₂O, 4:1, v/v) containing 0.2 mg/mL ribitol as an internal standard was added. A steel bead was placed into the tube, and the mixture was homogenized in a pre−cooled grinder at −20°C and 50Hz for 3min. Subsequently, 200μL of chloroform was added, followed by another 3min of grinding at 50Hz. The sample was then subjected to ultrasonic extraction for 30min and kept at −20°C for 30min. After low−temperature centrifugation (13,000×g, 4°C, 15min), the supernatant was transferred to a glass derivatization vial and dried under a gentle nitrogen stream. For oximation, 80 μL of 15 mg/mL methoxyamine hydrochloride in pyridine was added, the vial was vortexed for 2min, and incubated at 37°C in a shaking incubator for 90min. Thereafter, 80μL of N,O-bis(trimethylsilyl)trifluoroacetamide (BSTFA) containing 1% trimethylchlorosilane (TMCS) was added as the derivatization reagent. The mixture was vortexed for 2min and reacted at 70°C for 60 min. Finally, the vial was removed and allowed to equilibrate at room temperature for 30 min before GC−MS analysis.

The derived samples were analyzed on an Agilent 8890−5977B GC−MS system (Agilent Technologies, USA). Raw data files were processed using MassHunter Workstation Quantitative Analysis software (version 10.0.707.0) for peak picking, alignment, and other preprocessing steps. In the untargeted metabolomic workflow, metabolite identification required meeting at least two of the following three criteria: a high−resolution filter (HRF) score ≥80, a forward match similarity index (SI) ≥600, and a retention index difference (ΔRI) ≤50. Quantification was performed by normalizing the ion peak areas against the internal standard (ribitol), according to the formula:


$$\frac{\left[\text{Internalstandard}\right]}{\left[\text{Metabolite}\right]}=\frac{\text{Peakareaofinternalstandard}}{\text{Peakareaofmetabolite}}$$

This yielded a final data matrix of identified metabolites and their relative abundances.

Chromatographic conditions: After derivatization, samples were injected in split mode (split ratio 10:1) with an injection volume of 1 µL. Separation was achieved using a DB-5MS capillary column (40 m × 0.25 mm × 0.25 µm, Agilent 122-5532G), with the effluent directed to the mass spectrometer. The injector temperature was set at 300 °C. High-purity helium was used as the carrier gas at a constant flow rate of 1 mL/min. The solvent delay time was 5.5 min. Temperature program: The oven temperature was initially held at 60 °C for 0.5 min, then increased at a rate of 8 °C/min to 310 °C, and finally held at 310 °C for 6 min.

Mass spectrometry conditions: Ionization was performed using an electron impact (EI) source. The temperatures were set as follows: transfer line, 310 °C; ion source, 280 °C; and quadrupole, 150 °C. The electron energy was 70 eV. Data were acquired in full-scan mode over a mass range of “m/z” 35–550, with a scan rate of 3.2 scans per second.

#### 2.2.2 Routine chemical composition determination

The tobacco leaf samples were dried at 50 °C, destemmed, and ground. The contents of nicotine, total nitrogen, total sugar, reducing sugar, and chlorine were determined using continuous flow analysis according to the following industry standard methods: YC/T 159-2002, YC/T 161-2002, YC/T 160-2002, YC/T 173-2003, and YC/T 162-2002.

#### 2.2.3 Sensory quality evaluation

For sensory evaluation, the samples were destemmed, cut into shreds, dried, and conditioned to equilibrate moisture before being rolled into experimental cigarettes. In this study, we established quality indicators and a 9-point scoring standard for single-tobacco cigarette sensory evaluation with reference to the industry standard YC/T 138–1998 Tobacco and Tobacco Products. Sensory quality was assessed by a panel of 5 professional smoking experts from Hunan Tobacco Industry Co., Ltd., using a double-blind protocol. The evaluation covered seven key indicators: aroma quality, amount of aroma, miscellaneous gases, smoke concentration, momentum, mucosal irritation, and aftertaste, and the final score for each indicator was determined as the average value of all panelists’ independent ratings.

### 2.3 Data statistics and analysis

Principal component analysis (PCA) and partial least squares-discriminant analysis (PLS-DA) were performed. Differential metabolites were screened based on a combination of fold change (FC), p-value (*p* < 0.05), and variable importance in projection (VIP) values (VIP > 1). All figures were generated using the online platform provided by Majorbio Biotechnology Co., Ltd. All results are expressed as the standard error of the mean (SEM). Statistical analyses were performed using SPSS software, with significance assessed by one-way analysis of variance (ANOVA) followed by multiple comparisons. A statistical probability of *P* < 0.05 was considered statistically significant. Asterisks denote significant differences between groups, where *P* < 0.05 is indicated by “*” and *P* < 0.01 by “**”.

## 3 Results

### 3.1 Impacts of light qualities on sun-cured yellow tobacco quality

#### 3.1.1 Routine chemical composition analysis

As shown in (**Table 1**), supplemental lighting significantly altered the chemical composition of sun-cured tobacco leaves. Under NL treatment, the contents of total sugar, reducing sugar, chloride, total nitrogen, and nicotine were 14.7%, 10.4%, 0.416%, 2.51%, and 3.12%, respectively, yielding a sugar-to-nicotine ratio of 4.71 and a nitrogen-to-nicotine ratio (N/N) of 0.80.Compared to NL, the NL+R treatment induced more pronounced changes: significantly higher total sugar (15.8%), reducing sugar (12.2%), and chloride (0.457%) contents, alongside significantly lower total nitrogen (2.28%) and nicotine (2.83%) contents. Consequently, the sugar/nicotine ratio increased to 5.58 (N/N: 0.81), reflecting an optimized “increased sugar, decreased nicotine” profile. In contrast, the NL+B treatment showed no significant difference in total sugar (15.1%), reducing sugar (11.4%), or chloride (0.435%) content. However, total nitrogen (3.37%) and nicotine (4.20%) contents were significantly elevated. This resulted in a marked decrease in the sugar/nicotine ratio to 3.60 (N/N: 0.80), ultimately leading to a lower sugar/nicotine ratio than NL due to the effect of “increased nitrogen, elevated nicotine”.

**Table 1:** Tobacco chemical composition under different light quality treatments.

Sample	Total sugar (%)	Reducing sugar (%)	Chloride ion (%)	Total nitrogen (%)	Nicotine (%)	Sugar-to-nicotine ratio	Nitrogen-to-nicotine ratio
NL	14.7 ± 0.65	10.4 ± 0.35	0.416 ± 0.005	2.51 ± 0.12	3.12 ± 0.04	4.71	0.80
NL+R	15.8 ± 0.66*	12.2 ± 0.38*	0.457 ± 0.017*	2.28 ± 0.08*	2.83 ± 0.05*	5.58	0.81
NL+B	15.1 ± 0.61	11.4 ± 0.44	0.435 ± 0.005	3.37 ± 0.07*	4.20 ± 0.02*	3.60	0.80

“*” indicates a significant difference compared to NL (P<0.05). NL, Natural light treatment; NL+R, Natural light + red light treatment; NL+B, Natural light + blue light treatment. Data are presented as mean ± standard deviation (SD).

#### 3.1.2 Sensory quality evaluation

As shown in (**Table 2**), different supplemental lighting treatments had distinct effects on the sensory quality of sun-cured tobacco leaves. Under the NL treatment, the leaves received scores of 5.8 for aroma quality, 6.0 for both amount of aromay and momentum, and 6.5 for smoke concentration. Scores for miscellaneous gases, mucosa irritation, and aftertaste were all 5.5. Overall, the aroma quantity and strength were acceptable, but the aftertaste was slightly discomforting. Following the NL+R treatment, aroma quality improved to 6.2, while scores for miscellaneous gases, mucosa irritation, and aftertaste all increased to 6.0. Aroma quantity and smoke concentration remained at 6.0, while momentum was maintained at 5.5. In summary, this treatment enhanced aroma quality, reduced irritation, and provided a more comfortable aftertaste, although the overall sensory profile shifted somewhat toward that of typical flue-cured tobacco. In contrast, the NL+B treatment generally resulted in weaker sensory scores. Aroma quality, amount of aroma, miscellaneous gases, momentum, mucosa irritation, and aftertaste mostly scored 5.5, with only smoke concentration remaining at 6.0. The overall sensory profile was characterized by a pronounced lingering sensation on the tongue, slight discomfort, and a dry aftertaste. Based on these trends, red-light supplementation exerted a more pronounced optimizing effect on sensory quality, whereas blue-light supplementation somewhat diminished the comfort and harmony of the sensory experience.

**Table 2:** Sensory quality evaluation of sun-cured tobacco leaves.

Sample	Aroma quality	Amount of aroma	Miscellaneous gases	Smoke concentration	Momentum	Mucosa irritation	Aftertaste	Overall assessment
NL	5.8	6	5.5	6.5	6	5.5	5.5	The aroma intensity and impact were acceptable, but the aftertaste was slightly unsatisfactory.
NL+R	6.2	6	6	6	5.5	6	6	The distinctive style was attenuated, shifting toward an English-type flue-cured tobacco character, with improved aroma quality, reduced irritancy, and a more comfortable aftertaste.
NL+B	5.5	5.5	5.5	6	5.5	5.5	5.5	A pronounced tongue residue was noted, comfort was slightly compromised, and the aftertaste was dry.

NL, Natural light treatment; NL+R, Natural light + red light treatment; NL+B, atural light + blue light treatment.

### 3.2 Strategies for metabolomic data analysis and model evaluation

To clarify the specific effects of different supplemental lighting treatments on the metabolic characteristics of tobacco leaves under identical irradiation durations, and to avoid the dilution of differential signals that may arise from including too many groups in a full-set analysis, this study adopted a time-stratified analysis for partial least squares-discriminant analysis (PLS-DA). The analytical design used fixed irradiation duration as the key variable to eliminate interference from the natural metabolic background changes occurring during the curing process. The analysis specifically focused on the effects of light quality within the same time dimension. Based on the number of days of supplemental lighting (1, 2, 3, and 9 days), the 13 treatments were divided into four independent analysis groups: Group A (CK, NL+R1d, NL+B1d, NL1d; 1 day), Group B (CK, NL+R2d, NL+B2d, NL2d; 2 days), Group C (CK, NL+R3d, NL+B3d, NL3d; 3 days), and Group D (CK, NL+R9d, NL+B9d, NL9d; 9 days).Each group was designed to capture the metabolic differences induced by different light quality treatments under the same irradiation duration. This approach aimed to accurately identify the specific regulatory effects of light quality on tobacco leaf metabolism and to enhance the model’s discriminative accuracy for differential signals.

The PLS-DA models were validated with 200 permutation tests. The results revealed a clear trend, the explanatory power of the principal components increased progressively with longer supplemental lighting across the time-point groups. Specifically, in (**Supplementary Figure 2A**) (1 day), the first and second principal components (PC1 and PC2) explained 25.4% and 23.3% of the variance, respectively, with a cumulative total of 48.7%. In (**Supplementary Figure 2B**) (2 days), the values were 31.6% (PC1), 18.2% (PC2), and 49.8% (cumulative). A marked increase was observed in (**Supplementary Figure 2C**) (3 days), where PC1 explained 44.4% of the variance, PC2 11.3%, yielding a cumulative rate of 55.7%. This trend continued in (**Supplementary Figure 2D**) (9 days), with PC1 reaching 51.8%, PC2 8.33%, and a cumulative total of 60.13%.Notably, in the PLS-DA score plots for each group, samples from different light-quality treatments were clearly separated in space from the control (CK) samples. Furthermore, samples within each treatment group showed good clustering, with no significant outliers. These findings demonstrate that the time-stratified analysis effectively isolated the specific metabolic differences attributable to light quality at each time point by eliminating interference from varying treatment durations. The progressive increase in PC1’s explanatory power suggests that the regulatory effect of light quality on leaf metabolism intensifies with prolonged exposure. Consequently, the model’s discriminative power was consistently enhanced, providing a robust statistical foundation for the subsequent screening of differential metabolites and analysis of metabolic pathways.

### 3.3 Categories, pathways, and temporal dynamics of aroma-related differential metabolites

#### 3.3.1 Identification of aroma metabolites and screening of metabolic pathways

**Figure 1:**
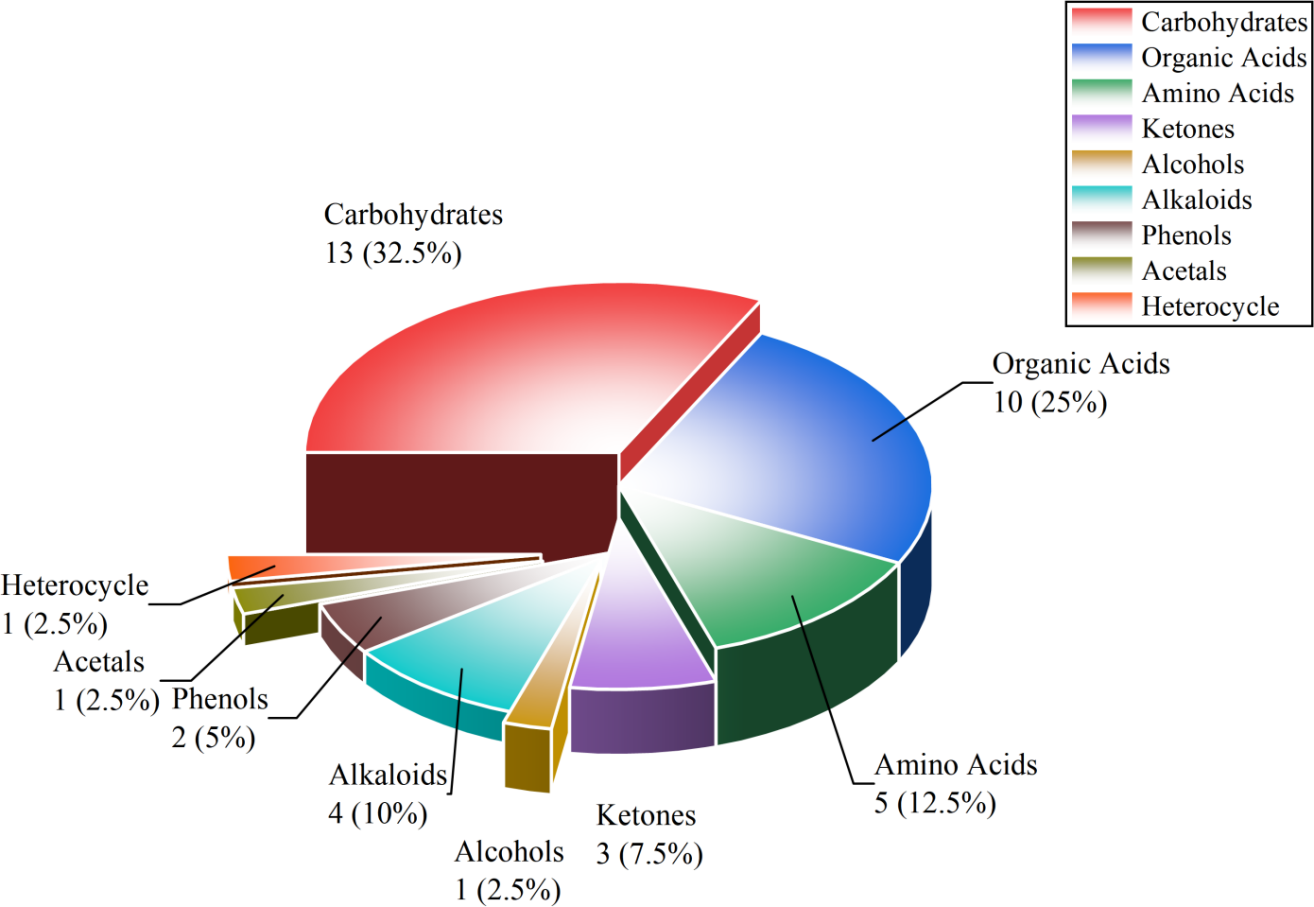
Category and proportion of differential aroma metabolites in tobacco leaves during curing. A total of 40 aroma-related differential metabolites were identified in this study, covering 9 categories. Carbohydrates (13 species, 32.5%); Organic acids (10 species, 25.0%); Amino acids (5 species, 12.5%); Alkaloids (4 species, 10.0%); Phenols (2 species, 5.0%); Ketones (3 species, 7.5%). Alcohols, Acetals, Heterocycles each accounted for 1 species (2.5% each).

In this study, differential metabolites were screened using thresholds of VIP > 1 and *p* < 0.05; this effectively excluded interfering metabolites and improved the accuracy and reliability of the screening results. Through metabolomic analysis, this study systematically clarified the dynamic variation rules of the metabolic network in sun-cured tobacco leaves during the curing process. Meanwhile, the pie chart (**Figure 1**) presents the categorical distribution and quantity proportion characteristics of aroma-related differential metabolites throughout the entire curing process. A total of 40 aroma-related differential metabolites were identified, covering 9 categories including carbohydrates, organic acids, and amino acids, and among these categories, carbohydrates (32.5%) and organic acids (25.0%) were the main differential categories, which suggests that the adjustment of core carbon metabolism is a universal characteristic during the curing process.

**Figure 2:**
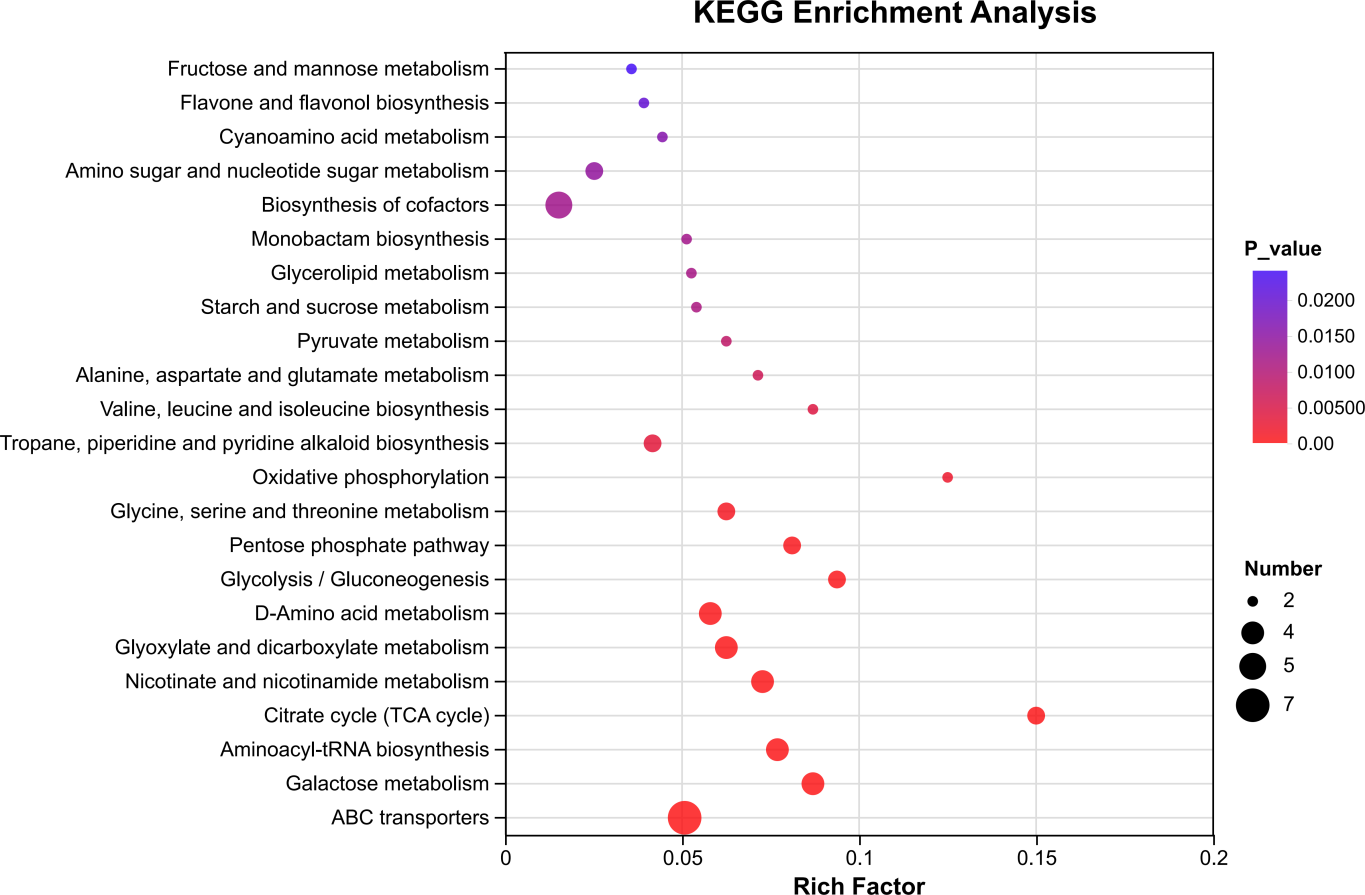
Differential metabolic pathways of tobacco leaf aroma during curing. Pathways were screened at a significance level of *P* < 0.05. The horizontal axis shows the Rich Factor, representing the enrichment level of differential metabolites in each pathway. The color of each dot corresponds to the enrichment P-value, with a gradient from purple to red indicating a decreasing P-value and thus increasing enrichment significance. Dot size reflects the number of differential metabolites mapped to each pathway, with larger dots denoting a greater number of metabolites.

Further, differential metabolic pathways with significant enrichment were screened in this study using a threshold of *P* < 0.05, resulting in the identification of 23 pathways in total. Analysis revealed that aroma-related differential metabolites were significantly enriched in a carbohydrate metabolism-centered network, which included key processes such as glycolysis/gluconeogenesis, the TCA cycle, and the pentose phosphate pathway (**Figure 2**; **Supplementary Table 1**). Among these enriched pathways, differential metabolites including glucose, D-glucopyranose, and mannose were enriched in multiple pathways, reflecting the extensive redistribution of carbon flux during the curing process. Meanwhile, involving metabolites such as fumaric acid, citric acid, L-serine, and L-threonine, the biosynthesis pathways of amino acids and secondary metabolites were also co-enriched.

#### 3.3.2 Temporal dynamics of aroma-related differential metabolites during curing

**Figure 3:**
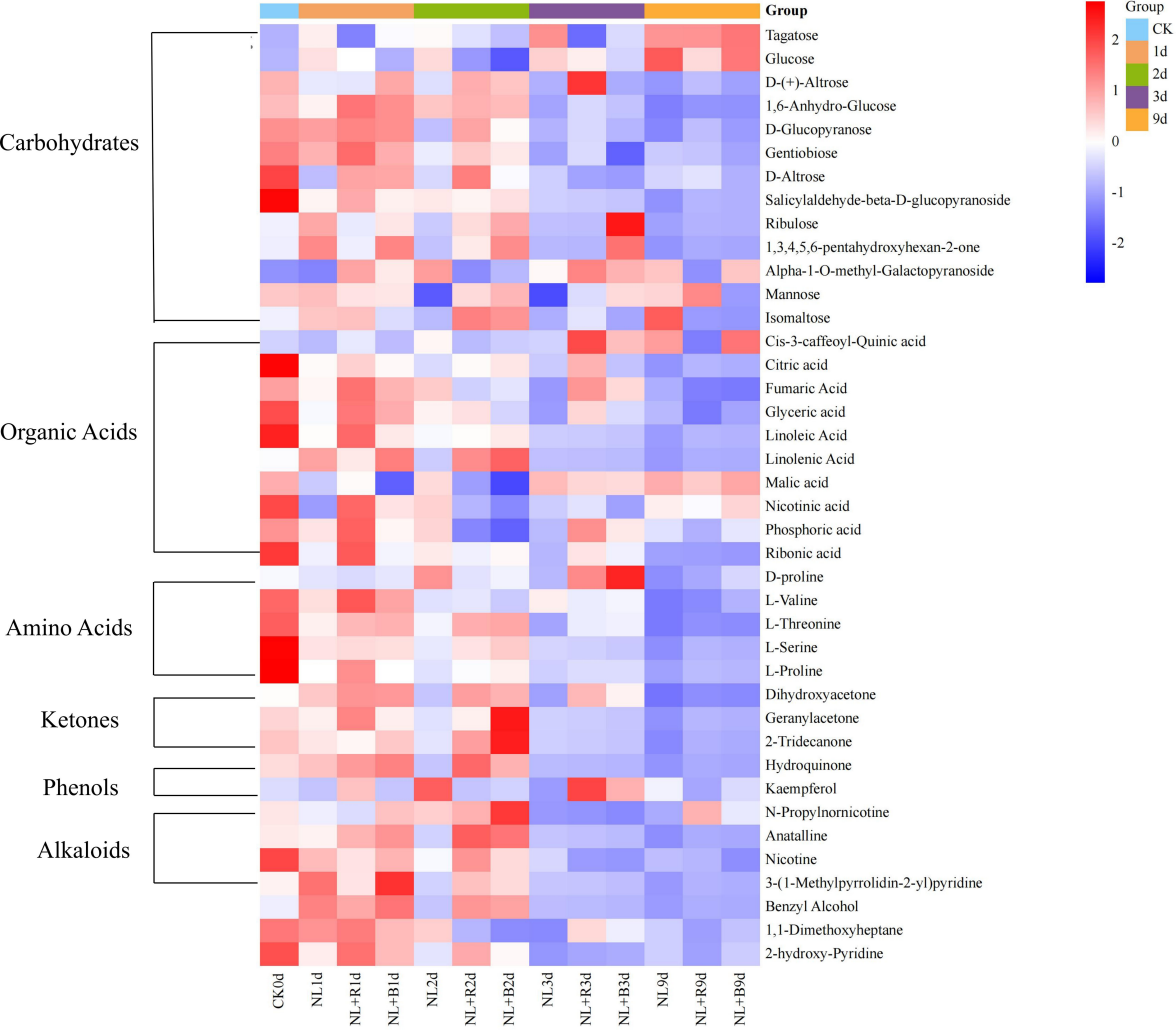
Cluster heatmap of differential aroma metabolites in tobacco leaves during Sun-curing. Heatmaps dynamic expression patterns of aroma-related differential metabolites under different light qualities during curing screening criteria VIP > 1 and *P* < 0.05 (*P* < 0.05 indicates statistical significance);Groups CK 0d (fresh tobacco leaves), 1d (the first day of curing), 2d (the second day of curing), 3d (the third day of curing), 9d (the ninth day of curing—the end of curing); The row represents individual metabolites, and the column displays samples. The rows also classify metabolites into distinct functional categories.

The heatmap in (**Figure 3**) displays the changing trends of aroma-related differential metabolites (screening criteria: VIP>1, *P* < 0.05) in different sun-cured tobacco samples across various curing days. Each column represents a specific curing day under a given light quality treatment, each row corresponds to one differential metabolite. The results indicate that metabolites from different classes exhibited distinct temporal dynamic patterns.

Aroma-related differential metabolites belonging to carbohydrates could be categorized into three typical expression patterns. The first pattern (e.g., glucose and tagatose) was characterized by suppression during the yellowing stage, followed by a sharp accumulation at the end of curing (day 9), which was particularly pronounced under the NL+R and NL+B treatments. This indicated that supplementary light qualities exerted a short-term inhibitory effect on the synthesis of these two metabolites; as the curing process progressed, metabolic activity gradually recovered, which might be associated with the carbon source supply demand for aroma compound synthesis in the later stage. The second pattern (e.g., D-glucopyranose and gentiobiose) featured high abundance in the early stage and a decrease in the later stage, with the expression levels in the NL+R and NL+B groups consistently higher than those in the NL group during the initial period. The third category of metabolites (e.g., mannose and isomaltose) exhibited treatment-specific fluctuations, suggesting that different light qualities exerted specific regulatory effects during the curing process.

Organic acids exhibited two opposite expression trends. The first trend (e.g., cis-3-caffeoylquinic acid, malic acid, nicotinic acid) was marked by substantial accumulation in the late stage of curing across all treatments. Among these three metabolites, the expression levels were higher in the NL 9d and NL+B 9d groups than in the NL+R 9d group. This indicated that different light quality treatments could regulate the synthesis and accumulation of such metabolites in sun-cured tobacco. The second trend featured fluctuating expression in the early stage and generally low expression in the late stage (e.g., fumaric acid, linoleic acid, and linolenic acid). For fumaric acid, linoleic acid, and linolenic acid, the duration of high expression was longer in the NL+R and NL+B groups than in the NL group. This suggested that supplementary light could delay the consumption of some organic acid substrates to a certain extent. Notably, most amino acids, ketones, phenols, and alkaloids exhibited an overall decreasing trend.

Therefore, the temporal dynamics of aroma-related differential metabolites clearly indicate that, compared with natural light treatment, supplementary red and blue light can exert differential regulation on the variation timing and intensity of core metabolites.

### 3.4 Metabolite characteristics and regulatory mechanisms specific to light quality

#### 3.4.1 Comparison of aroma difference metabolites of different light quality treatments

**Figure 4:**
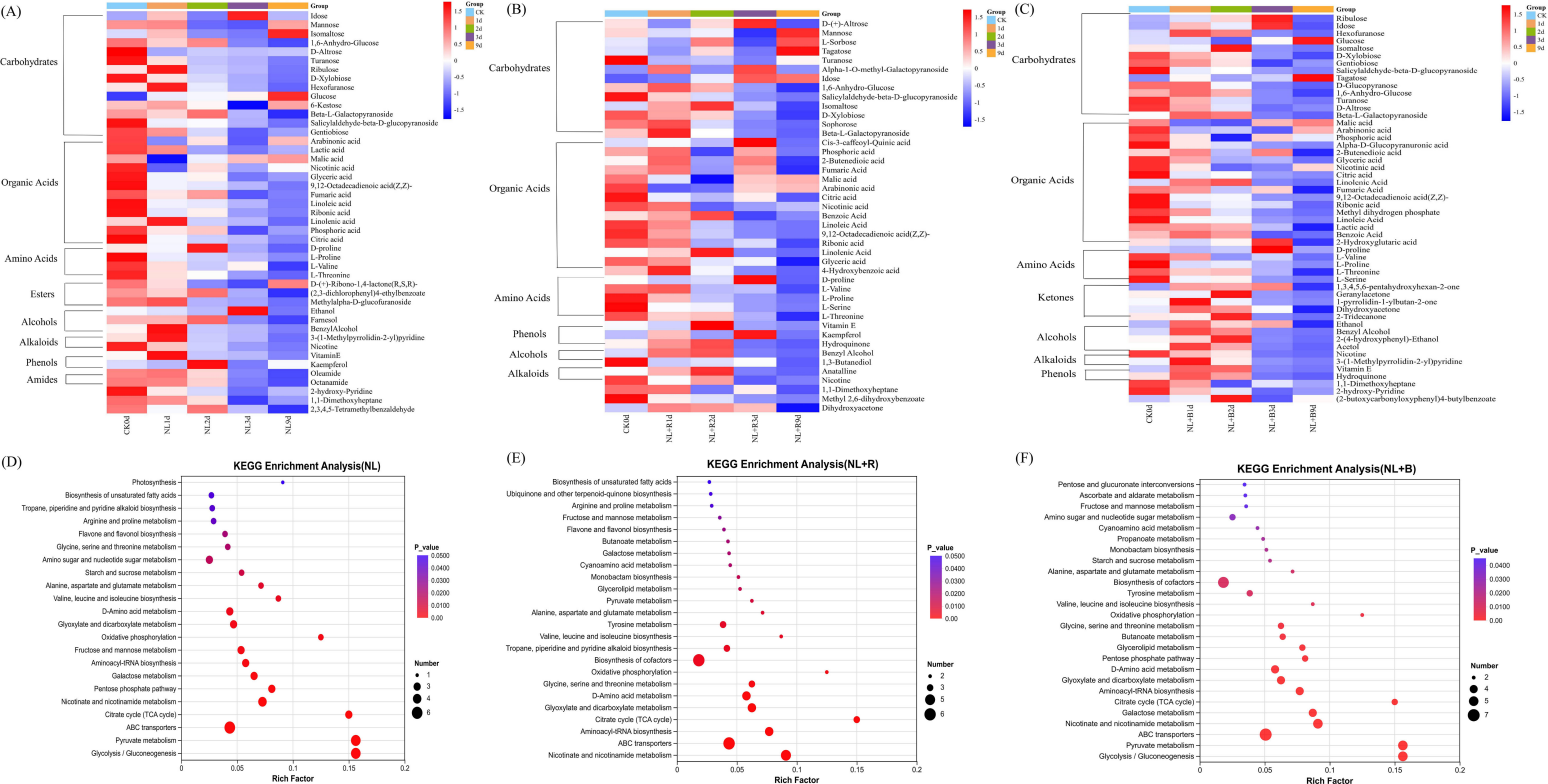
Heatmaps of aroma-related differential metabolites and KEGG pathway enrichment analysis in sun-cured tobacco leaves under different light quality treatments. Heatmaps dynamic expression patterns of aroma-related differential metabolites during curing screening criteria VIP > 1 and *P* < 0.05 (*P* < 0.05 indicates statistical significance): **(A)** Natural light (NL) treatment; **(B)** Natural light + red light (NL+R) treatment; **(C)** Natural light + blue light (NL+B) treatment; Groups CK 0d (fresh tobacco leaves), 1d (the first day of curing), 2d (the second day of curing), 3d (the third day of curing), 9d (the ninth day of curing—the end of curing); The row represents individual metabolites, and the column displays samples. The rows also classify metabolites into distinct functional categories. Metabolites significantly decreased were displayed in blue, while metabolites significantly increased were displayed in red. The KEGG enrichment bubble plots significantly enriched metabolic pathways screening criterion *P* < 0.05 (*P* < 0.05 indicates statistical significance):(D) NL treatment; **(E)** NL+R treatment; **(F)** NL+B treatment; Horizontal axis Rich Factor; the color of dots corresponds to the enrichment significance (P-value); the size of dots represents the number of differential metabolites.

To elucidate the differential regulatory effects of the three light quality treatments (NL, NL+R, and NL+B) on the metabolome during sun-cured tobacco leaf curing, this study conducted a systematic analysis of aroma differential metabolites across key curing time points (i.e., CK 0d and days 1, 2, 3, and 9). By employing heatmap analysis (using the CK group as the baseline), we aimed to reveal the specific regulatory impacts and dynamic metabolic characteristics induced by each light treatment (**Figures 4A–C**).

A common basic metabolic framework shared by all treatments was observed in this study. This framework encompasses aroma-related differential metabolites derived from primary metabolisms such as carbohydrates, organic acids, and amino acids, and the contents of these metabolites exhibited the most significant changes on the first day of curing. The variation trends of secondary metabolites including phenols, alcohols, and alkaloids also began to emerge on the first or second day of curing. Beneath this common metabolic framework, the regulatory specificity of different light quality treatments became particularly evident, primarily in the accumulation of aroma components and their temporal dynamics.

Compared with the stable and low-fluctuation metabolic profile of the NL group, both the NL+R and NL+B combined light quality treatments significantly slowed down the overall variation trend of metabolites. On the 3rd day of curing, the expression levels of most metabolites in the NL group had already dropped to relatively low levels (**Figure 4A**). In contrast, the NL+R group not only maintained higher expression levels of some metabolites than the NL+B group, but also had a richer variety of highly expressed metabolites (**Figure 4B**). This observation suggests that red light may activate more diverse metabolic pathways in the mid-stage of curing. In terms of aroma component accumulation, the differences in the accumulation of aroma-related differential metabolites on the 9th day of curing were directly linked to the flavor differentiation of tobacco leaves. Among them, the NL group mainly accumulated common sugars such as mannose and glucose, reflecting the stable state of basic metabolic processes in the tobacco leaves of this group. In comparison, the NL+R group specifically accumulated certain rare or functional sugars such as tagatose and idose, indicating that red light has the potential to promote tobacco leaves to form more complex flavor characteristics. The NL+B group only detected two types of sugars, mannose and tagatose, with not only a relatively single accumulation type but also lower overall expression levels than the NL+R group (**Figure 4C**); however, this group exhibited distinct characteristics in organic acid metabolism. Further analysis showed that the NL and NL+R groups mainly accumulated metabolites such as arabinonic acid and malic acid, which continued the characteristics of stable basic metabolism. In contrast, the NL+B group additionally showed high expression levels of nicotinic acid on this basis, which also became a prominent feature of the organic acid metabolism pattern of this group.

#### 3.4.2 Different light qualities regulate metabolic pathways

##### 3.4.2.1 Common characteristics

The three light treatments (NL, NL+R, and NL+B) exhibited significantly different regulatory effects on the plant metabolic network (**Figures 4D–F**). At the threshold of *P* < 0.05, the NL+B group had the largest number of differential metabolic pathways (Rai et al., 2025), followed by the NL+R group (Mantle et al., 2024). These results indicated that supplementary light treatments exerted more pronounced perturbation effects on the metabolic network, and distinct preferences existed among different groups in regulating core metabolic pathways such as carbohydrate metabolism and amino acid metabolism. Notably, the NL+R and NL+B treatments co-enriched five pathways, namely butanoate metabolism, biosynthesis of cofactors, monobactam biosynthesis, glycerolipid metabolism, and tyrosine metabolism. This finding reflected the shared role of supplementary light treatments in driving the metabolic network toward more in-depth secondary metabolism.

##### 3.4.2.2 Specific sexual characteristics

In the metabolic pathways with P<0.05 significance, analysis of light-specific regulation pathways revealed that different light conditions specifically regulate distinct metabolic pathways.

Under NL treatment, inorganic phosphate (Pi) was significantly enriched in the photosynthetic pathway. As a key substrate for ATP synthesis, the accumulation of Pi can enhance the catalytic efficiency of ATP synthase during the light reactions, promote efficient ATP production, and thereby provide ample energy support for dark reaction processes such as the Calvin cycle-mediated carbon fixation. This mechanism helps maintain the efficient operation of photosynthetic carbon assimilation (**Supplementary Figure 3**).

Under the NL+R treatment, vitamin E and 4-hydroxybenzoic acid were identified as the core, enriched differential metabolites within the Ubiquinone and other terpenoid-quinone biosynthesis pathway (**Supplementary Figure 4**). Their biosynthetic pathways and metabolic fluxes are as follows: Vitamin E biosynthesis uses 2-Methyl-6-phytylquinol as the common precursor, which then proceeds through two parallel branches to synthesize the final product, vitamin E. There are three pathways for 4-Hydroxybenzoic acid biosynthesis. Pathway 1 uses 4-Hydroxybenzoyl-CoA as the precursor; the target compound is directly generated via thioester bond hydrolysis and is ultimately converted to shikonin. Pathway 2 starts with chorismate as the precursor; the target compound is produced under the catalysis of lyase and subsequently participates in the biosynthesis of ubiquinone and ubiquinone-n. Pathway 3 takes the target compound as the starting material, and its recycling is achieved through glycosylation and deglycosylation.

In the NL+B treatment group, the glycolysis/gluconeogenesis pathway was significantly enriched with metabolites including D-glucopyranose, lactic acid, ethanol, α-D-glucose, and β-D-glucose (**Supplementary Figure 5**); among these, the synthesis of α-D-glucose originated from two branch pathways of α-D-glucose 1-phosphate in the starch and sucrose metabolism pathway, ultimately generating lactic acid and ethanol—the characteristic end products of this pathway.

Notably, the NL+B treatment not only regulated the core carbohydrate metabolic pathways but also exerted a significant impact on the propanoate metabolism pathway. In this pathway, lactic acid and acetol were identified as the key enriched metabolites (**Supplementary Figure 6**). Analysis of their biosynthetic pathways showed that the accumulation of lactic acid was derived from two metabolic inputs. The synthesis of acetol was mainly associated with the glycolytic shunt metabolism. Dihydroxyacetone phosphate (Glycerone-P), generated via glycolysis, was cleaved to form methylglyoxal under the catalysis of methylglyoxal synthase. Subsequently, methylglyoxal underwent sequential catalysis by methylglyoxal reductase and NADP-dependent alcohol dehydrogenase and was ultimately reduced to acetol through reduction reactions.

### 3.5 Association analysis of metabolite changes and tobacco quality

In this study, by integrating chemical composition, sensory evaluation, and metabolomics data, we systematically elucidated the elaborate mechanism through which light quality regulation influences the final tobacco leaf quality by reshaping specific metabolic pathways. For the NL+R treatment, metabolomic analyses revealed that it specifically activated the ubiquinone and terpenoid-quinone biosynthesis pathways at the metabolic level, promoting the conversion of 4-hydroxybenzoic acid into antioxidant substances such as vitamin E, and accumulating rare sugars including tagatose and idose. This metabolic characteristic was consistent with the results of conventional chemical composition analysis, which showed a significant increase in sugar content and notably higher total sugar and reducing sugar levels than those in the NL treatment. Together, they formed a more coordinated chemical foundation for tobacco leaf flavor development. Among these components, antioxidants help reduce the harshness and mucosa irritation of tobacco smoke (Zong et al., 2023), while specific sugars act as key precursors for the formation of a sweet, mellow taste and smooth aftertaste (Li J. et al., 2024). This collectively explains the optimized sensory evaluation results of this treatment, including improved aroma quality, reduced off-notes and irritation, and enhanced aftertaste. In contrast, the NL+B treatment drove a drastic shift in metabolic flux toward glycolysis/gluconeogenesis and propanoate metabolism pathways, leading to the rapid decomposition of sugars and substantial production of end products such as lactic acid, ethanol, and acetol. This metabolic alteration was consistent with its conventional chemical composition features, namely the significant increase in total nitrogen and nicotine contents. The accumulated acidic and irritating metabolites directly disrupted the acid-base balance and component coordination of tobacco leaves (Chen et al., 2024), resulting in sensory evaluation outcomes characterized by strong smoke potency alongside distinct residual sensation on the tongue, dry aftertaste, and poor smoking comfort. By comparison, the NL treatment maintained stable basic photosynthetic carbon metabolism, with both its metabolite profile and quality performance remaining at the baseline level. Collectively, light quality determines the accumulation ratio of key flavor precursors and harmful byproducts through the precise regulation of primary metabolic flux direction and secondary metabolic activation degree, thereby ultimately governing the coordination of chemical components and the quality of sensory attributes of tobacco leaves.

## 4 Discussion

The curing stage of sun-cured tobacco is a decisive period for its quality formation, and light quality, as a core environmental regulatory factor during this process, can influence the shaping of final sensory characteristics by interfering with the chemical composition of tobacco leaves. In this study, three light treatments (NL, NL+R, and NL+B) were set up to systematically explore the regulatory effects of light quality on the chemical composition, sensory quality, and metabolic dynamics of tobacco leaves. The specific action mechanisms of different light qualities were clarified, which provides experimental basis and theoretical support for the precise regulation of sun-cured tobacco quality.

As a key environmental signal, light quality exerts its regulatory effect starting from the plant photoreceptor system (Bao et al., 2024), and ultimately manifests itself in the specific remodeling of central carbon metabolic nodes. Metabolic pathway enrichment analysis in this study indicated that the differential metabolites under different light quality treatments were all significantly enriched in core hub pathways such as glycolysis/gluconeogenesis, the TCA cycle, and the pentose phosphate pathway. This is consistent with the general understanding that light quality extensively modulates plant primary metabolism (Zhao et al., 2024).However, different light qualities induced directional divergences in this common metabolic network. The NL+R treatment specifically activated the biosynthetic pathways of ubiquinone and terpenoid quinone. This pathway is closely associated with cellular redox homeostasis and antioxidant capacity (Mantle et al., 2024); its activation usually implies that the generation and utilization of reducing power (NADPH) are enhanced, which creates favorable conditions for downstream reductive biosynthesis (e.g., carbohydrates and antioxidants) (Guo et al., 2025).In contrast, the NL+B treatment strongly enriched the glycolysis/gluconeogenesis and propionate metabolic pathways, leading to the massive accumulation of fermentation end products such as lactic acid and ethanol. This phenomenon aligns with studies showing that blue light promotes respiratory metabolism and induces metabolic changes in some plants, which may result from the direct or indirect regulation of related enzyme activities by blue light signals (Bao et al., 2024).Meanwhile, the enrichment of phosphate in the photosynthetic pathway under the NL treatment directly reflects the state of maintaining basic photosynthetic carbon assimilation efficiency under natural light. These results demonstrate that different light qualities regulate the key nodes of carbon metabolism through differentiated signal transduction at the initial stage of curing, thereby setting the initial direction of subsequent metabolic flux.

Based on the regulation of specific metabolic nodes, the limited carbon skeletons and energy flux inside cells undergo competitive redistribution, namely the bias of metabolic flux toward different end products. The time-stratified dynamic data and terminal metabolome profiles in this study clearly delineated two opposing metabolic patterns. In contrast, the NL+R treatment directed metabolic flux toward synthesis and storage. This was specifically reflected by the preferential accumulation of rare sugars such as tagatose and idose. This phenomenon not only enriched the diversity of the sugar pool in tobacco leaves, but also served as a more stable form of carbon reserve or a precursor of flavor compounds (Rai et al., 2025). Meanwhile, the synthesis level of vitamin E was significantly increased. This biosynthetic pathway is closely linked to the generation of reducing power during photosynthesis, and the enhancement of its synthesis suggested that red light treatment might optimize the subsequent allocation and utilization efficiency of photosynthetic assimilation products. This pattern of promoting sugar accumulation and enhancing antioxidant capacity was consistent with the research trend (Jia et al., 2024; Pashkovskiy et al., 2024) that red light increases the contents of carbohydrates and antioxidants in fruits and vegetable^s^ (Hu et al., 2024).On the contrary, the NL+B treatment shifted metabolic flux toward catabolism and fermentation. Glycolysis not only accelerated the consumption of substrates such as glucose (Rinehart et al., 2018), but also channeled a large amount of carbon flux into the biosynthetic pathways of fermentation products including lactic acid, ethanol and acetol. This phenomenon resembled some metabolic characteristics of plants under hypoxia or stress conditions. In the meantime, this intense catabolism provided abundant carbon skeletons and ATP for nitrogen assimilation. This provided metabolic-level support for the enhancement of nitrogen metabolism, which was consistent with studies showing that blue light generally promotes nitrogen uptake and assimilation in plants.

The competitive outcomes of metabolic flux are directly reflected as quantifiable differences in macroscopic chemical compositions. The synthesis-and-storage metabolic flux induced by NL+R treatment ultimately shaped a favorable chemical ratio characterized by high sugar and medium-to-low alkaloid contents. This result accurately verified the application of the “carbon-nitrogen metabolic balance” theory in tobacco quality. When carbon flux was preferentially directed toward sugar synthesis, the carbon skeletons and energy allocated to alkaloid biosynthesis became relatively limited (Chen et al., 2025; Hay Mele et al., 2025), thus optimizing the sugar-to-alkaloid ratio. In contrast, the catabolism-and-fermentation metabolic flux under NL+B treatment resulted in an unbalanced chemical profile with relatively low sugar, high alkaloid, and high organic acid contents. Among these changes, the significant increase in nicotine was a combined effect of blue light-enhanced carbon and nitrogen metabolism (Morańska et al., 2023). The accumulation of organic acids served as a direct indicator of active fermentative metabolism, implying potential alterations in the intracellular pH environment (Yoon et al., 2024).The chemical composition under NL treatment maintained a relatively balanced baseline state. Collectively, these three sets of data formed a distinct gradient, visually demonstrating that light quality shapes a continuous spectrum of chemical compositions by regulating metabolic flux.

The final sensory quality is the result of chemical components acting synergistically on the human sensory system. The association among metabolic regulation, chemical composition, and sensory attributes established in this study exhibits clear interpretability and predictability. The balanced chemical profile induced by NL+R treatment inevitably led to a superior sensory experience. High sugar content provided the material basis for the production of sweet and mellow sensations (Carstens and Carstens, 2022). Antioxidants such as vitamin E might reduce the generation of free radicals or irritating carbonyl compounds in cigarette smoke, thereby alleviating harshness and irritation (Bao et al., 2024). An appropriate nicotine level ensured smoking satisfaction while avoiding acrid and pungent sensations. For these reasons, this treatment achieved the highest scores in flavor quality and aftertaste comfort during sensory evaluation. In contrast, the chemical drawbacks of NL+B treatment were directly translated into poor sensory performance. Excessive short-chain acids such as lactic acid imparted intense acidic irritation and persistent residual sensations (Barroso et al., 2024). Excessively high nicotine levels exacerbated the harshness and irritation of smoke strength. Meanwhile, the relative lack of sugar made the smoke deficient in sweet and smooth buffering effects, highlighting a dry sensation (Ganz et al., 2022). The low scores of all its sensory indicators were the comprehensive reflection of these negative effects. These findings directly correlated the previously ambiguous sensory descriptions with objective metabolite and chemical composition data, providing a basis for predicting tobacco sensory quality using metabolomics.

In summary, this study clarifies the specific mechanisms by which different light qualities regulate the quality of sun-cured tobacco. These findings not only provide solid theoretical support for the field of tobacco metabolism and quality regulation but also offer operable practical guidance for the precise quality improvement of sun-cured tobacco through light environment regulation in production. In the future, further research can explore the interaction between light quality and other environmental factors such as temperature and humidity, and develop efficient and economical comprehensive regulation schemes, so as to contribute to the sustainable development of the tobacco industry.

## 5 Conclusion

This study investigated the effects of light quality on the quality and metabolism of sun-cured tobacco during curing. The results demonstrated that the NL+R treatment optimized the sugar-nicotine ratio by increasing sugar and reducing alkaloid content, which enhanced aroma quality, lowered irritation, and improved overall sensory performance. In contrast, the NL+B treatment led to nitrogen accumulation and elevated alkaloids, resulting in an imbalanced sugar-nicotine ratio and detectable sensory defects. The NL treatment maintained only moderate quality. At the metabolic level, carbohydrates and organic acids together represented over half of the detected metabolites, with carbon metabolism and amino acid metabolism being the predominant pathways. Distinct aroma different metabolite classes showed characteristic temporal dynamics throughout curing. In terms of significantly altered metabolic networks (*p* < 0.05), the NL treatment sustained basal metabolism and specifically regulated photosynthesis. The NL+R treatment activated multiple pathways, delayed metabolic decline, promoted the accumulation of rare sugars, and upregulated the ubiquinone and terpenoid-quinone biosynthesis pathways. The NL+B treatment regulated glycolysis/gluconeogenesis and propanoate metabolism, suppressed carbohydrate synthesis, and stimulated the accumulation of nitrogen-containing compounds. In summary, compared to the traditional curing approach that depends on variable natural conditions, supplementing specific light qualities can achieve targeted improvement of tobacco leaf quality. This is of significant practical importance for overcoming the long-term constraints imposed by the natural environment on sun-cured tobacco production. Our findings provide a key scientific basis for shifting the sun-cured tobacco industry from passive reliance on natural conditions to active, precise, and standardized production management based on light environment regulation. Future studies could further explore the interactions between light quality and other environmental factors to refine comprehensive control strategies.

## Data Availability

The original contributions presented in the study are included in the article/**Supplementary Material**. Further inquiries can be directed to the corresponding authors.
