# Supplementary material for: Deciphering the mechanism of light quality regulating the quality of sun-cured yellow tobacco based on GC-MS non-targeted metabolomics
Source: Front Plant Sci. 2026 Apr 22;17:1770112. doi: 10.3389/fpls.2026.1770112 (PMC13170478; doi:10.3389/fpls.2026.1770112)
Supplement: Supplementary file 1 [file DataSheet1.docx]

Table S1. Details of differential metabolic pathways in tobacco leaves during curing

| Second Category | Pathway description | Enriched metabolites |
| --- | --- | --- |
| Carbohydrate metabolism | Fructose and mannose metabolism | Mannose、Glucose |
| Global and overview maps | Biosynthesis of cofactors | L-Valine、L-Serine、Nicotinic acid、Citric acid、Vitamin E |
| Biosynthesis of other secondary metabolites | Flavone and flavonol biosynthesis | Kaempferol |
| Carbohydrate metabolism | Amino sugar and nucleotide sugar metabolism | Mannose、Glucose、D-Glucopyranose |
| Metabolism of other amino acids | Cyanoamino acid metabolism | Mannose、Glucose、D-Glucopyranose |
| Biosynthesis of other secondary metabolites | Monobactam biosynthesis | L-Serine、 L-Threonine |
| Lipid metabolism | Glycerolipid metabolism | Glyceric acid、Dihydroxyacetone |
| Carbohydrate metabolism | Starch and sucrose metabolism | Glucose、Isomaltose、D-Glucopyranose |
| Carbohydrate metabolism | Pyruvate metabolism | Fumaric acid、Malic acid |
| Amino acid metabolism | Alanine, aspartate and glutamate metabolism | Fumaric acid、Citric acid |
| Biosynthesis of other secondary metabolites | Tropane, piperidine and pyridine alkaloid biosynthesis | Nicotinic acid、Nicotine、3-(1-Methylpyrrolidin-2-yl)pyridine、Anatabine |
| Amino acid metabolism | Valine, leucine and isoleucine biosynthesis | L-Valine、 L-Threonine |
| Energy metabolism | Oxidative phosphorylation | Phosphoric acid、Fumaric acid |
| Amino acid metabolism | Glycine, serine and threonine metabolism | L-Serine、 L-Threonine、Glyceric acid |
| Carbohydrate metabolism | Pentose phosphate pathway | Glyceric acid、Glucose、D-Glucopyranose |
| Carbohydrate metabolism | Glycolysis / Gluconeogenesis | Glucose、D-Glucopyranose |
| Metabolism of other amino acids | D-Amino acid metabolism | L-Serine、 L-Threonine、D-Proline、L-Proline |
| Carbohydrate metabolism | Glyoxylate and dicarboxylate metabolism | L-Serine、Glyceric acid、Malic acid、Citric acid |
| Metabolism of cofactors and vitamins | Nicotinate and nicotinamide metabolism | Nicotinic acid、Fumaric acid、Nicotine、3-(1-Methylpyrrolidin-2-yl)pyridine |
| Translation | Aminoacyl-tRNA biosynthesis | L-Valine、L-Serine、 L-Threonine、L-Proline |
| Carbohydrate metabolism | Citrate cycle (TCA cycle) | Fumaric acid、Malic acid、Citric acid |
| Carbohydrate metabolism | Galactose metabolism | Tagatose、 Mannose、Glucose、D-Glucopyranose |
| Membrane transport | ABC transporters | L-Valine、L-Serine、Phosphoric acid、 L-Threonine、L-Proline、 Mannose、Glucose、D-Glucopyranose |


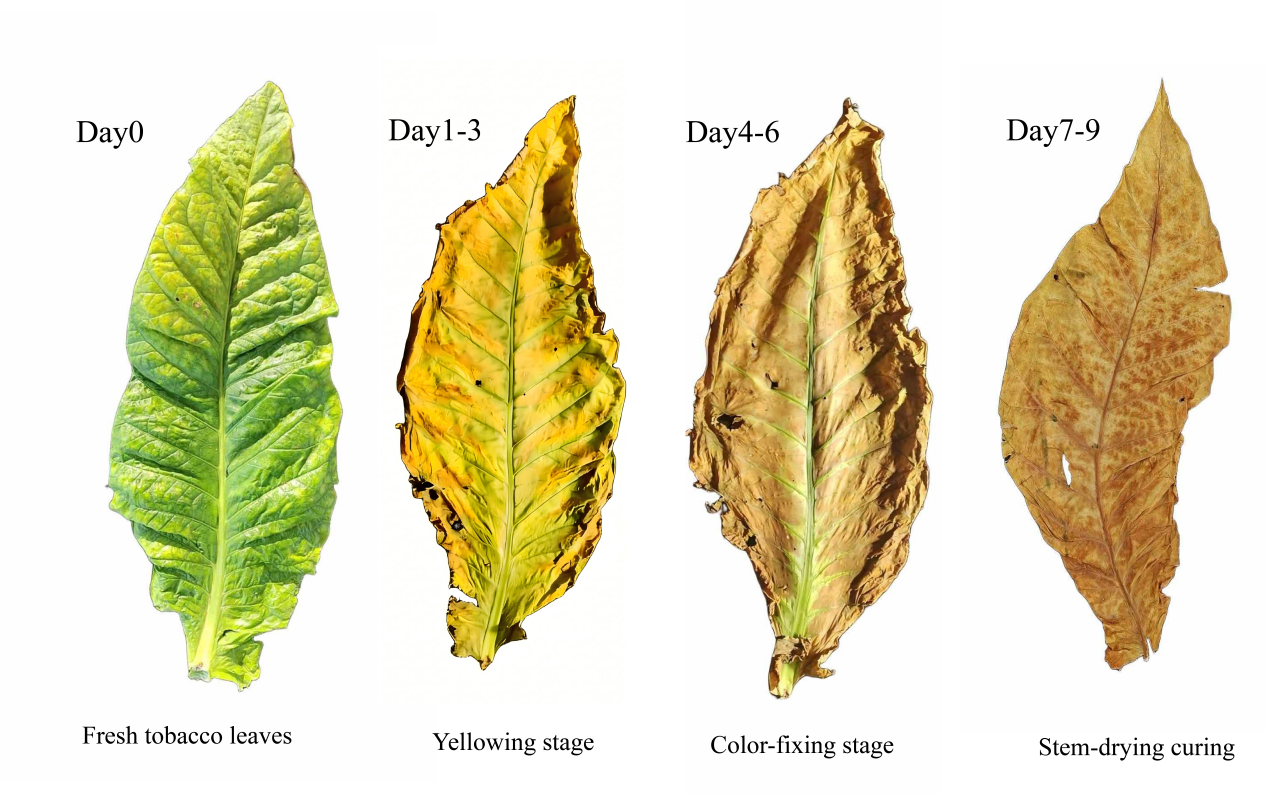


**Figure S1.**Schematic diagram of the complete tobacco leaf drying cycle

Note：During the curing process, tobacco leaves go through four stages: Fresh Leaves (Day 0) → Yellowing Stage (Days 1-3) → Color-Fixing Stage (Days 4-6) → Stem-Drying Stage (Days 7-9)


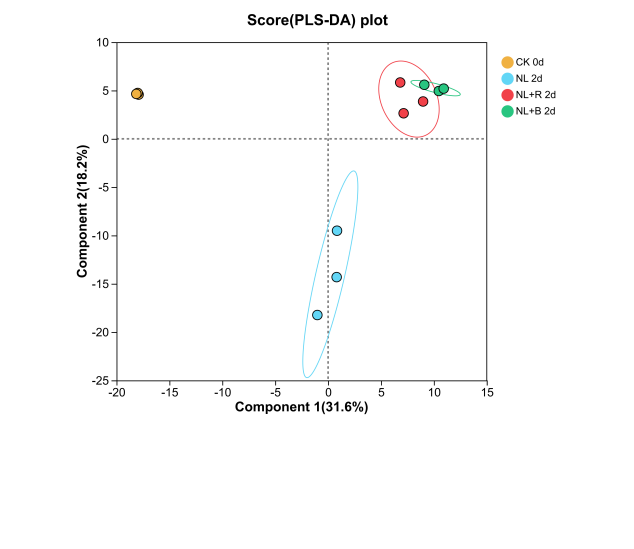

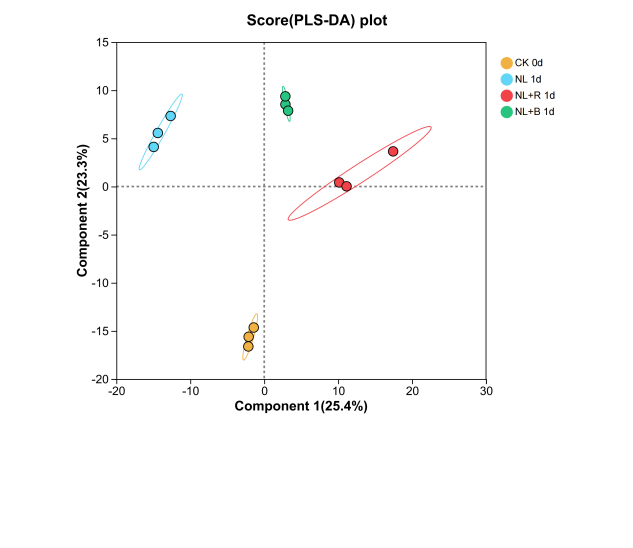


(B)

(A)


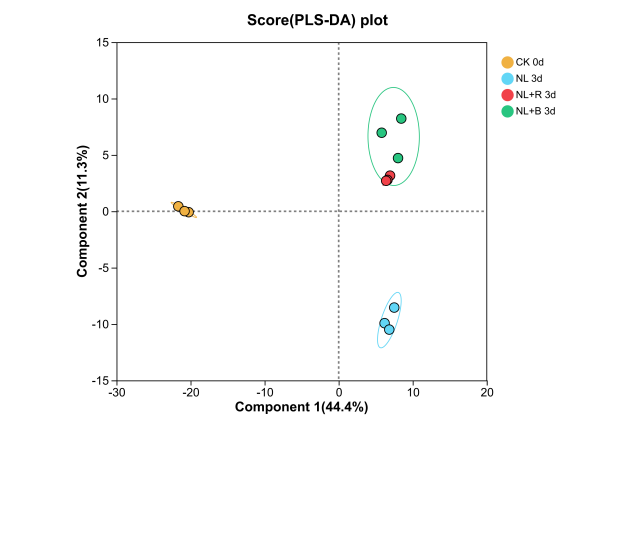

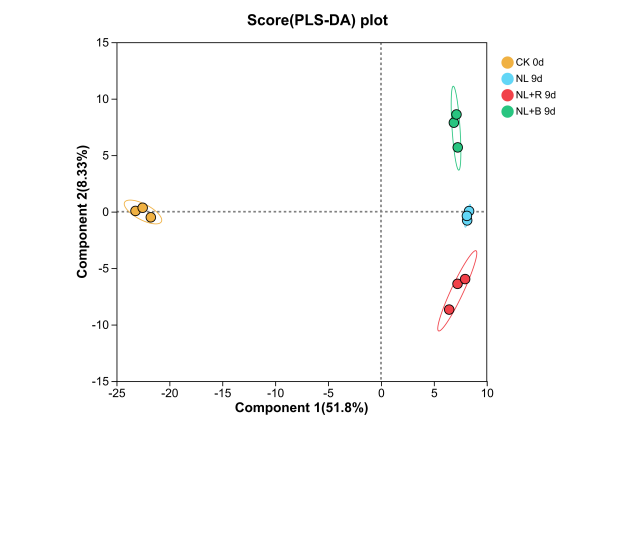


(D)

(C)

Figure S2. PLS-DA score plot of the tobacco leaf metabolome under different light supplementation treatments.

Note：CK0d represents the initial control group (fresh, un-cured leaves). Each subplot corresponds to the curing duration of sun-cured yellow tobacco under different light qualities:(A) Day 1; (B) Day 2; (C) Day 3; (D) Day 9.All subplots show the separation of sun-cured yellow tobacco samples treated with different light qualities at different curing time points. Each point in the figure represents an individual sample, and different colors represent distinct experimental groups.


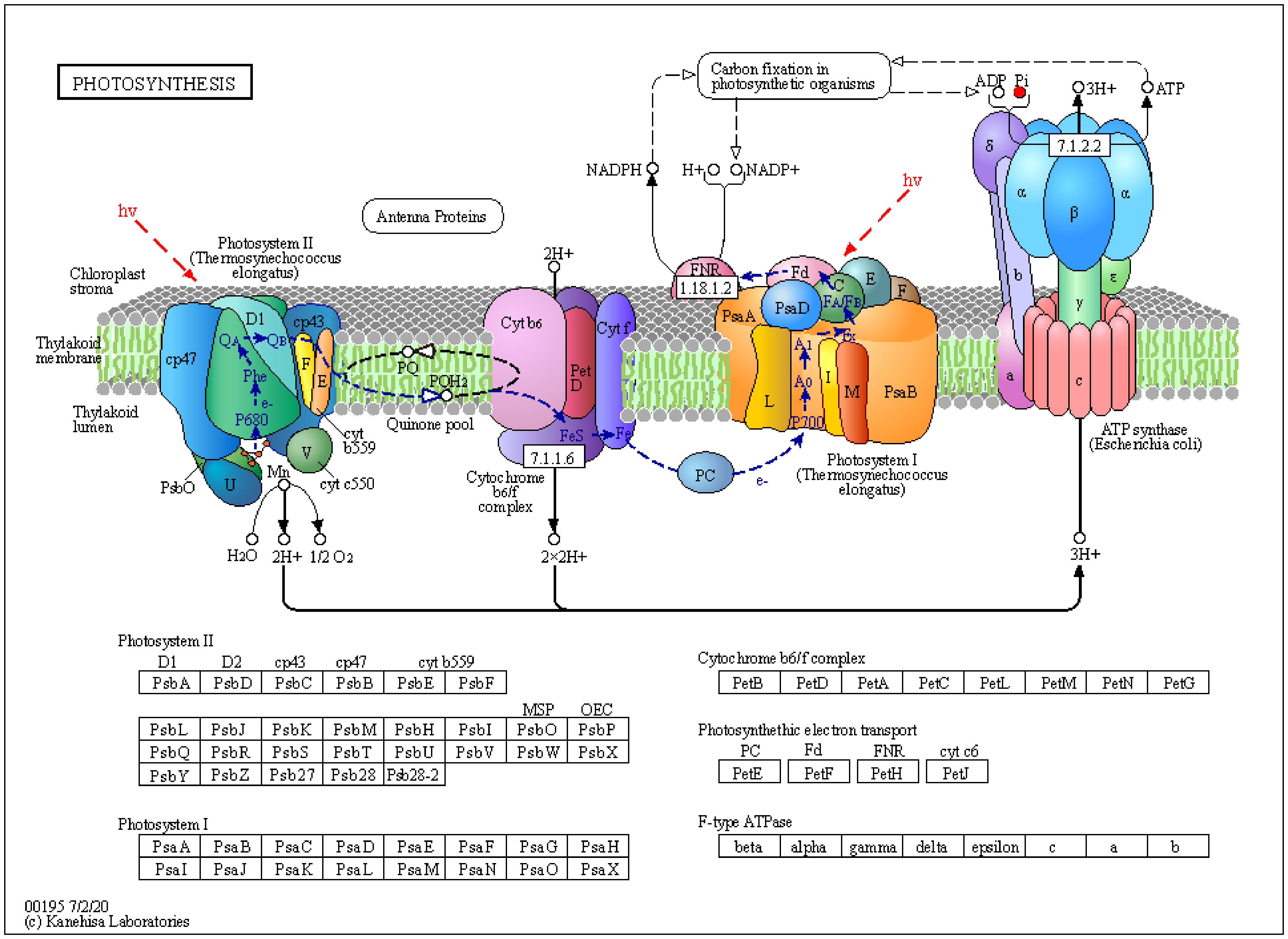


Figure S3.Photosynthesis and differential metabolites under NL treatment

Note:Specific metabolic pathways obtained in NL compared to NL+R and NL+B. Red dots represent metabolites enriched in the pathways.


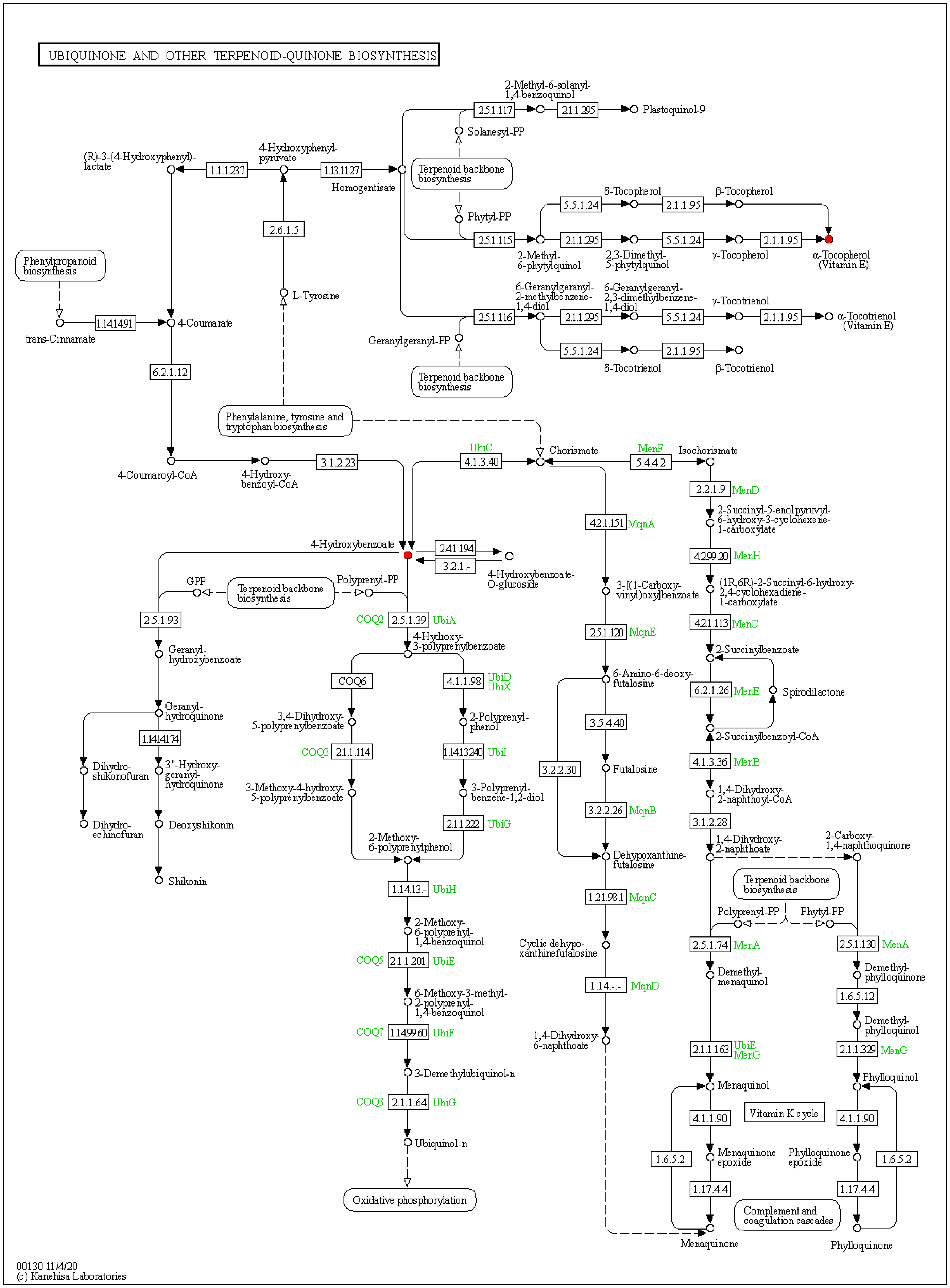


Figure S4.Ubiquinone and other terpenoid-quinone biosynthesis and differential metabolites under NL+R treatment

Note:Specific metabolic pathways obtained in NL+R compared to NL and NL+B. Red dots represent metabolites enriched in the pathways.


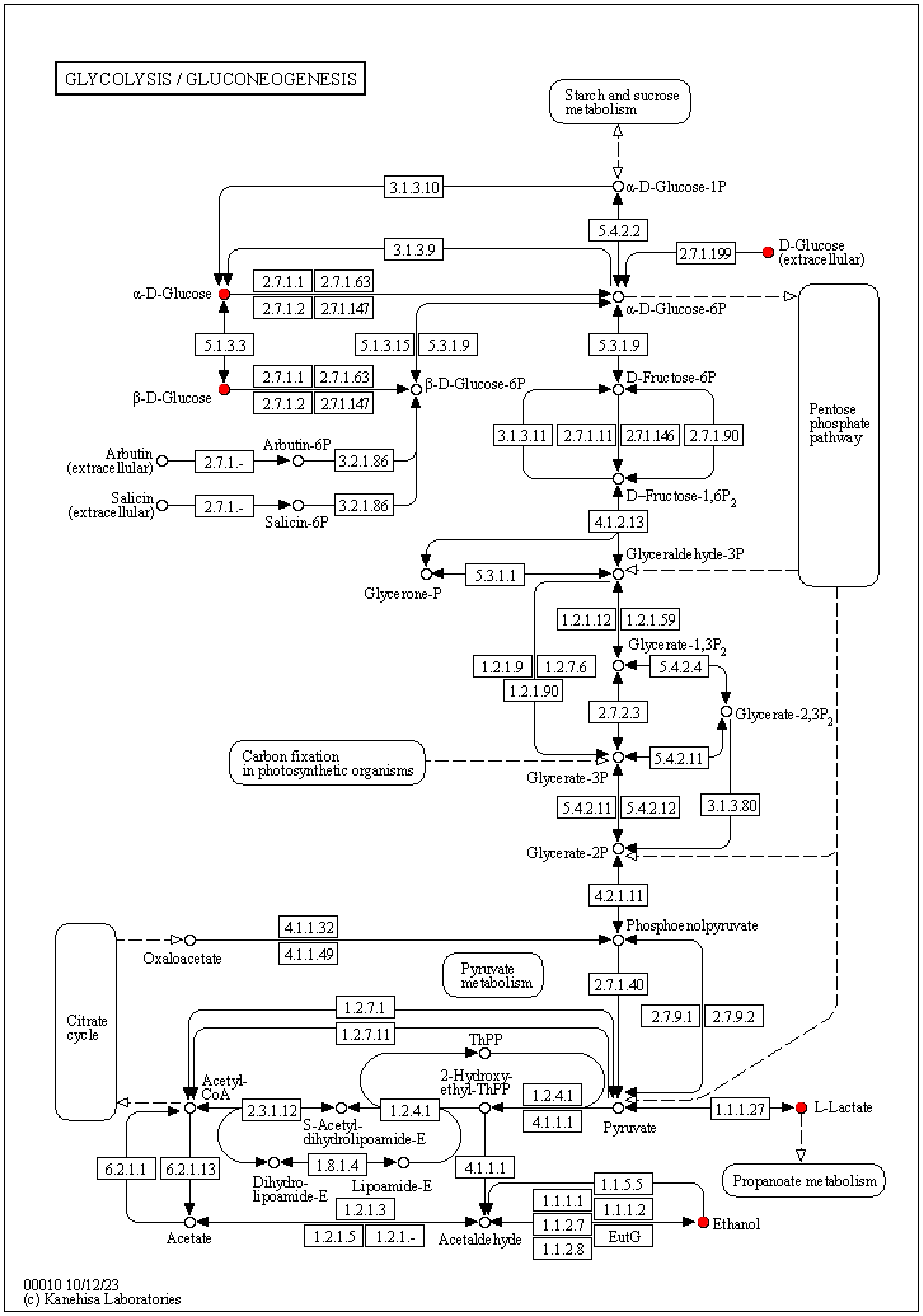


Figure S5. Glycolysis/gluconeogenesis pathway and differential metabolites under NL+B treatment

Note:Specific metabolic pathways obtained in NL+B compared to NL and NL+R. Red dots represent metabolites enriched in the pathways.


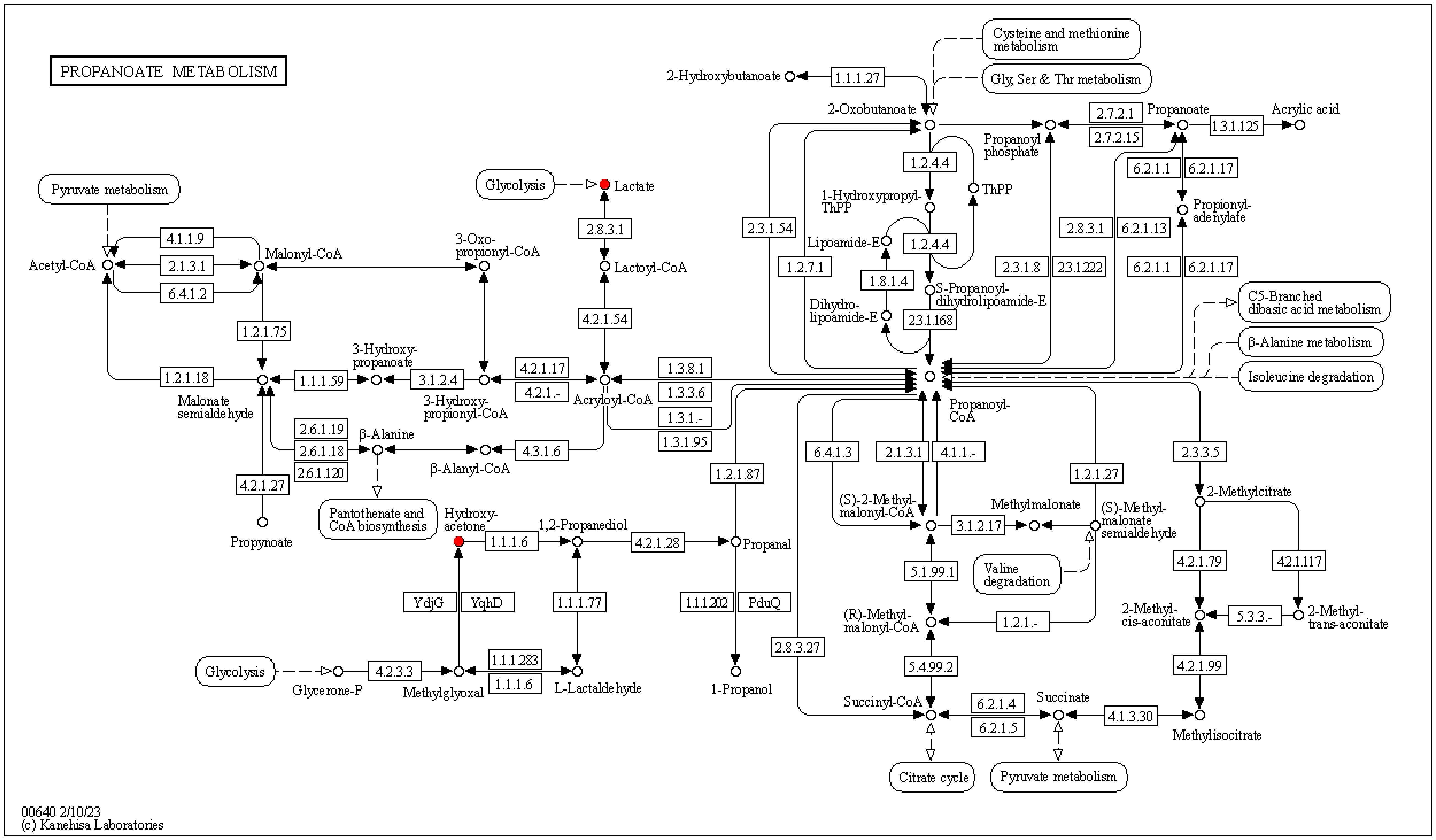


Figure S6.Propanoate metabolism and differential metabolites under NL+B treatment.

Note:Specific metabolic pathways obtained in NL+B compared to NL and NL+R. Red dots represent metabolites enriched in the pathways.
